# microRNA-155, Induced by Interleukin-1ß, Represses the Expression of Microphthalmia-Associated Transcription Factor (MITF-M) in Melanoma Cells

**DOI:** 10.1371/journal.pone.0122517

**Published:** 2015-04-08

**Authors:** Nathalie Arts, Stefania Cané, Marc Hennequart, Juliette Lamy, Guido Bommer, Benoît Van den Eynde, Etienne De Plaen

**Affiliations:** 1 Ludwig Institute for Cancer Research, Brussels and Université Catholique de Louvain, Brussels, Belgium; 2 de Duve Institute, Université Catholique de Louvain, Brussels, Belgium; University of Connecticut Health Center, UNITED STATES

## Abstract

Loss of expression of surface antigens represents a significant problem for cancer immunotherapy. Microphthalmia-associated transcription factor (MITF-M) regulates melanocyte fate by driving expression of many differentiation genes, whose protein products can be recognized by cytolytic T lymphocytes. We previously reported that interleukin-1ß (IL-1ß) can downregulate MITF-M levels. Here we show that downregulation of MITF-M expression by IL-1ß was paralleled by an upregulation of miR-155 expression in four melanoma lines. We confirmed that miR-155 was able to target endogenous MITF-M in melanoma cells and demonstrated a role for miR-155 in the IL-1ß-induced repression of MITF-M by using an antagomiR. Notably, we also observed a strong negative correlation between MITF-M and miR-155 levels in a mouse model of melanoma. Taken together, our results indicate that MITF-M downregulation by inflammatory stimuli might be partly due to miR-155 upregulation. This could represent a novel mechanism of melanoma immune escape in an inflammatory microenvironment.

## Introduction

The *Microphthalmia-associated transcription factor* (*MITF*) gene generates several transcripts and proteins through the use of different promoters and alternative splicing of the first exon [1]. The isoform MITF-M is expressed exclusively in melanocytes and is a key transcription factor that regulates many genes involved in survival, proliferation and differentiation [2]. In melanoma cells, MITF-M regulates the expression of differentiation genes like *TYR (tyrosinase)*, *MLANA (Melan-A/Mart-1)* or *SILV (pMel17/gp100)*. Their protein products are shared with normal melanocytes and can be recognized by specific cytolytic T lymphocytes (CTL) at the surface of the tumor cells [3] [4] [5] [6]. At first sight, it might seem surprising that differentiation antigens would be considered as targets of immunotherapy. However, some data indicate that loss of the expression of Melan-A in a melanoma metastasis favors the escape of this tumor from the immune response [7].

Unfortunately, even when anti-tumor T lymphocytes are present, the majority of metastatic melanomas are still progressing. Changes on the side of the CTLs and on the side of the tumor cells, as well as their crosstalk contribute to this observation (reviewed in [8]).

On the CTL side, immunosuppressive cells such as activated regulatory T cells (Tregs) in the tumor microenvironment can lead to anergy [9]. In addition, many tumors produce enzymes that degrade tryptophan, thereby depriving the T lymphocytes of an essential amino acid [10] [11]. On the tumor site, expression of the tumor antigen can be compromised at several levels that include mutations in the gene encoding the antigenic peptide, loss of the expression of an HLA allele or a mutation in the gene encoding ß-2 microglobulin [8]. Last but not least, melanoma cells might lose expression of the protein from which the CTL-targets are derived. From a therapeutic standpoint, it is therefore of considerable interest to understand how the expression of potential CTL-targets is regulated. Understanding these mechanisms might potentially allow a therapeutic intervention that will lead to increased expression of CTL-targets. In the present study, we will focus our attention on the regulation of MITF-M and its target genes by the inflammatory microenvironment.

Cytokines play a crucial role in the immune response and modulate several pathways [12]. In the tumor microenvironment, they have a significant influence on the progression of the tumor and the anti-tumor immune response [12]. It has been shown that Interferon-γ (IFN-γ) represses the expression of Melan-A, Tyrosinase–related protein 1 (TRP-1) and gp100 in melanoma cells and that this repression reduced recognition of these cells by specific anti-Melan-A CTL [13]. IFN-γ can be produced by activated T lymphocytes or natural killer cells in the tumor stroma [14]. In addition, other cytokines like Tumor Necrosis Factor-α (TNF-α), secreted by activated T lymphocytes or macrophages, can be induced at the tumor site. TNF-α was reported to inhibit the expression of tyrosinase in B16 melanoma cells [15]. Moreover, the study of adoptive cell transfer therapies (ACT) with specific anti-gp100 CTLs in mice showed that some melanoma cells became resistant to the lysis induced by these CTLs thanks to a repression in the expression of gp100 [16]. This inhibition was induced by the presence of TNF-α in an inflammatory environment [16]. Another inflammatory cytokine, IL-1, was shown to be produced by melanoma cells themselves but its effect on melanogenesis is still not well understood [14] [17] [18].

For this reason, we had previously investigated the effect of IL-1ß on melanoma cell lines. Several melanoma cell lines showed a reduced expression of MITF-M when they were incubated with IL-1ß [19]. This repression was associated with a downregulation in the expression of differentiation antigens and led to a decreased recognition of melanoma cells by CTLs directed against those antigens, suggesting that this could represent a novel mechanism of tumor immune escape. We also discovered that this repression was Nuclear Factor-κB (NF-κB) and c-Jun N-terminal kinase (JNK)-dependent but we were not able to identify further mechanistic links between IL-1ß and the reduction of the expression of MITF-M.

The characterization of the mechanistic link between IL-1ß and MITF-M downregulation is the topic of the present study.

## Materials and Methods

### Cell lines, tissue samples, media and reagents

Melanoma cell lines were established in our laboratory [19], except for SK-MEL-23 received from Dr E. Stockert (Memorial Sloan-Kettering Cancer Center, New York) [20]. They were grown in Iscove’s modified Dulbecco’s medium (IMDM) (Invitrogen Life technologies) complemented with 10% fetal bovine serum (HyClone), 0.55 mM L-arginine, 0.24 mM L-asparagine, 1.5 mM L-glutamine, 100 U/ml penicillin, and 100 μg/ml streptomycin at 37°C in a humidified atmosphere containing 8% CO_2_. Melanoma cells were treated with IL-1ß at 10 ng/ml (PeproTech).

The mouse model of inducible melanoma has been created and used in our laboratory. This model has been approved by the local ethics committee. The head of this committee is Professor Jean-Paul Dehoux (Faculty of Medicine, Université Catholique de Louvain). Mice bearing tumors were anesthetized in a cage with CO_2_ and then killed by cervical dislocation. Tumor samples were collected by excision and directly frozen at -80°C in optimal cutting temperature (OCT) compound. The same day, slides of 10 μm were made from those samples and 10 slides of each sample were used to extract total RNA.

### Quantitative RT-PCR for mRNAs

RNA samples from cell lines and tissues were extracted with the TriPure Isolation Reagent (Roche) and the Nucleospin RNA kit (Macherey-Nagel), respectively. Total RNA (including small RNAs such as miRNAs) was incubated with TURBO DNAse (Turbo DNA-free kit, Ambion). 2 μg of total RNA was then used to synthesize cDNA with the RevertAid reverse transcriptase (Thermo Scientific) according to the manufacturer’s protocol. Quantitative reverse transcription (RT)-polymerase chain reaction (PCR) amplifications were done with the PCR core kit (Eurogentec) and with the use of 1/40 of the reaction volume from the reverse transcription in an ABI Prism 7300 Real Time PCR system (Applied Biosystems). The PCR primers (Eurogentec) used were: *human MITF-M sense* 5’-GGAATTATAGAAAGTAGAGGGA-3’ and *human MITF-M antisense* 5’-ACATGGCAAGCTCAGGAC-3’; *human tyrosinase sense* 5’- CCAGCATCATTCTTCTCCTCTTG-3’ and *human tyrosinase antisense* 5’-GTGGACTAGCAAATCCTTCCAG-3’; *human ß-actin sense* 5’-GGCATCGTGATGGACTCCG-3’ and *human ß-actin antisense* 5’-GCTGGAAGGTGGACAGCGA-3’; *murine MITF-M sense* 5’-AGGAGGACTAAGTGGTCTGCG-3’ and *murine MITF-M antisense* 5’-CCCTGGTTGCTGTAGAGGTCG-3’; *murine tyrosinase sense* 5’-CTAACTTACTCAGCCCAGCATC-3’ and *murine tyrosinase antisense* 5’-GGGTTTTGGCTTTGTCATGG-3’; *murine IL-1ß sense* 5’-ACAGGCTCCGAGATGAACAA-3’ and *murine IL-1ß antisense* 5’-TTGCTTGGGATCCACACTCTC-3’; *murine ß-actin sense* 5’-CTCTGGCTCCTAGCACCATGAAG-3’ and *murine ß-actin antisense* 5’- GCTGGAAGGTGGACAGTGAG-3’. The 5’-FAM/3’-TAMRA specific labeled probes (Eurogentec) used were: *human MITF-M* 5’-CCACCTCGAAAACCCCACCAAGTAC-3’; *human tyrosinase* 5’-CTGTTGTACTCCTCCAATCGGCTACAG-3’; *human ß-actin* 5’-TCAAGATCATTGCTCCTCCTGAGCGC-3’; *murine MITF-M* 5’-TCCGGCCTTGCAAATGGCAAATAC-3’; *murine tyrosinase* 5’-CTCCTCCTGGCAGATCATTTGTAGCAGATCAGA-3’, *murine IL-1ß* 5’-CTGTCGGACCCATATGAGCTGAAAGCTCTC-3’, *murine ß-actin* 5’-ATCGGTGGCTCCATCCTGGC-3’. Samples for quantitative PCR were assayed in duplicate and expression levels were normalized to ß-actin.

Statistical analyzes were performed using the Student’s t-test with a p-value of less than 0.05 (double-sided) considered as statistically significant (p-value < 0.05 was annotated *; < 0.01 **; <0.001 ***).

### Quantitative RT-PCR for miRNAs

Specific miRNAs were reverse transcribed from 10 ng of total RNA and analyzed by PCR on the ABI 7300 Real Time PCR System using the corresponding TaqMan MicroRNA Assay (Applied Biosystems). Results were normalized to the expression of RNU44 in human samples and of U6 in murine samples (TaqMan MicroRNA Assays, Applied Biosystems). The assay IDs used were: hsa-miR-96 (#000434), hsa-miR-137 (#001129), hsa-miR-148a (#000470), hsa-miR-155 (#002623), hsa-miR-182 (#002334), hsa-miR-340 (#002258), human RNU-44 (#001094), mmu-miR-155 (#002571), murine U6 (#001973).

### Luciferase reporter assays

The 3kb 3’UTR of MITF-M was amplified by PCR from LB2259-MEL cDNA and cloned downstream of the Renilla luciferase gene into the psiCHECK-2 plasmid (Promega). A mutation of 7 nucleotides in the putative binding site of miR-155 was introduced using the QuickChange II XL Site-Directed Mutagenesis Kit (Agilent Technologies). HEK-293T cells were then cotransfected with 0.65 μg of the reporter plasmid and 10 nM of miRNAs mimics (Applied Biosystems) with Lipofectamine 2000 Transfection Reagent (Life Technologies) in a 12 well-plate. Renilla and Firefly luciferase activities were assessed 24 hours after the transfection using the Dual-Luciferase Reporter Assay System (Promega).

### Western Blot

Total protein lysates from cell lines were obtained by homogenization of cells in lysis buffer [0.1% sodium dodecyl sulfate, 1% sodium deoxycholate, 0.5% NP-40 in PBS] with a protease inhibitor cocktail (Complete Mini, Roche). Proteins samples were subjected to electrophoresis on NuPage 4–12% Bis-Tris gels (Invitrogen Life technologies) using MOPS/SDS running buffer, with NuPage antioxidant added to the inner chamber, according to the manufacturer’s protocol (XCell Sure Lock). Proteins were transferred from the gel to a nitrocellulose membrane using the iBlot Cell Transfer System (Invitrogen). The membranes were blocked in PBS containing 5% dried milk and 0.1% Tween-20 for 1 hour, and then incubated at 4°C overnight with the following primary antibodies diluted in PBS containing 2% BSA and 0.1% Tween-20: anti-MITF rabbit monoclonal antibody #12590 (1:1,000 dilution; Cell Signaling), anti-tyrosinase mouse monoclonal antibody (T311) sc-20035 (1:1,000 dilution; Santa Cruz Biotechnology), anti-ß-actin mouse monoclonal antibody clone AC-15 A5441 (1:10,000 dilution; Sigma-Aldrich). After washing of the membranes in PBS containing 0.1% Tween-20, they were incubated with the following secondary antibodies in PBS containing 5% dried milk and 0.1% Tween-20: anti-mouse IgG-HRP #HAF007 (1:1,000 dilution for tyrosinase and 1:15,000 dilution for ß-actin; R&D systems) or anti-rabbit IgG-HRP sc-2313 (1:5,000 dilution; Santa Cruz Biotechnology). Membranes were incubated with the SuperSignal West Pico or West Femto Chemiluminescence Substrates (Thermo Fisher Scientific) and exposed to Hyperfilm ECL (GE healthcare).

### Transfections with mimics or antagomirs

LB2201-MEL cells were transfected with 30 nM of miR-155 mimic (Applied Biosystems) with Lipofectamine 2000 Transfection Reagent (Life Technologies) using the manufacturer’s protocol. Cells were collected after 24h or 48h directly in TriPure Isolation Reagent (Roche) for mRNA analyzes and in lysis buffer for protein analyzes. LB2201-MEL cells were transfected with 150 nM of a miR-155 specific Anti-miR miRNA Inhibitor (#AM17000, Applied Biosystem) or a control inhibitor using 1 μl per well of Lipofectamine 2000 Transfection Reagent (Life Technologies) in a final volume of 500 μl in a 12-well plate.

### Tirp Mice Model

TiRP-10B;Ink4a/Arf^flox/flox^ mice that were named “Tirp mice”were previously described [21].

### Supplementary data

#### Quantitative RT-PCR

Quantitative RT-PCR in the intron 1 of MITF-M was performed with the primers: *MITF-M intron 1 sense* 5’- AAGAGACGTATAGTTAATTAGCAG-3’ and *MITF-M intron 1 antisense* 5’- GGCTCTTCCTTCAGGCACAAACAT-3’. The *MITF-M intron 1* probe was: 5’- ATTCTGTTGGTGGCCTGGTCAGTTTC-3’. The primers and probe for the *MITF-M inter-exon* assay are those described in materials and methods.

Quantitative RT-PCR for PAX3 and SOX10 were performed with the primers and probes: PAX3 (Hs00240950_m1) (Life Technologies) and SOX10 (Hs00366918_m1) (Life Technologies).

#### Western Blot

The additional antibodies used were: anti-PAX3 mouse monoclonal antibody (1:500 dilution; R&D Systems) and anti-SOX10 rabbit monoclonal antibody (1:1,000 dilution; Abcam).

## Results

### IL-1ß concomitantly upregulates miR-155 and downregulates MITF-M in melanoma cell lines

**Fig 1:**
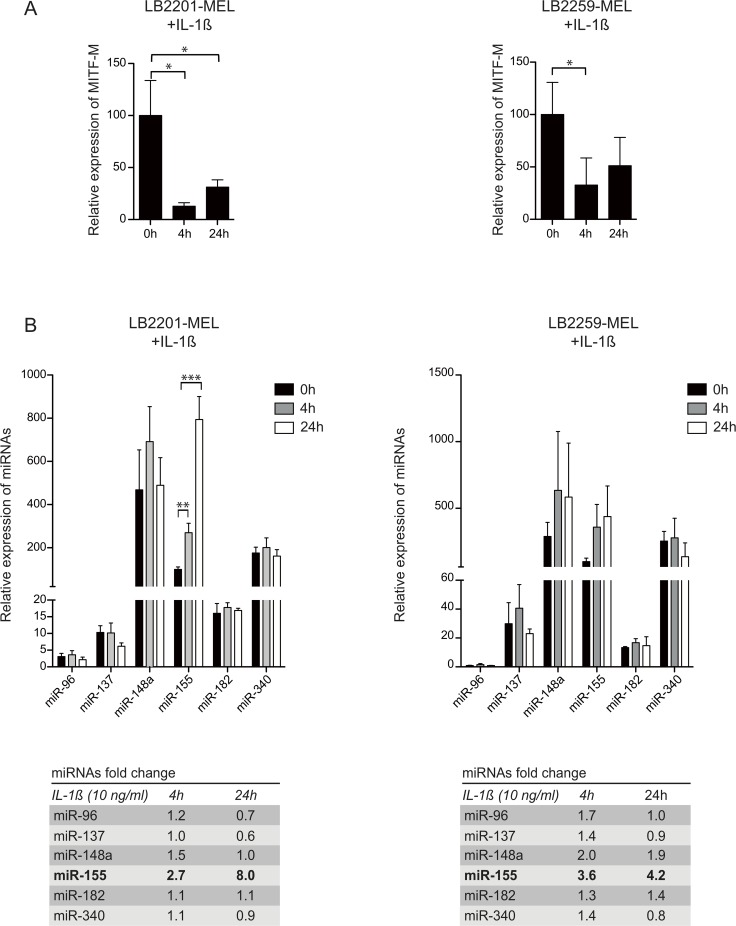
IL-1ß downregulates the expression of MITF-M and upregulates the expression of miR-155 in 2 melanoma cell lines. (**A**) Expression of MITF-M was analyzed by quantitative RT-PCR in melanoma cell lines LB2201-MEL and LB2259-MEL incubated with IL-1ß (10 ng/ml) for 4h or 24h. The result was normalized to ß-actin expression (means ± SD for 3 or 4 independent experiments, respectively). (**B**) Expression of miRNAs potentially targeting MITF-M (miR-96, miR-137, miR-148a, miR-155, miR-182 and miR-340) was analyzed by quantitative RT-PCR in the same samples and normalized to RNU44 expression. The table indicates the miRNA fold change when cells were treated with IL-1ß for 4h or 24h.

When analyzing the expression of MITF-M in two melanoma cell lines, LB2201-MEL and LB2259-MEL, we observed a significant downregulation of the expression of MITF-M mRNA after incubation with IL-1ß for 4h and 24h, consistent with a previous study from our laboratory (Fig 1A) [19]. This could be due either to reduced production (e.g. via repression of MITF-M transcription) or to destabilization of the MITF-M mRNA.

We first investigated the possibility that IL-1ß induces the expression of miRNAs targeting and destabilizing MITF-M transcripts. Several miRNAs have been described to target MITF. miR-137, miR-148 and miR-340 were shown to repress MITF-M expression in melanoma cells [22] [23] [24]. Additionally, MITF was identified as a target for miR-96 and miR-182 in the mouse retinal pigmented epithelium [25]. Finally, upregulation of miR-155 in monocytes was shown to play a role in repressing MITF expression, thereby favoring the acquisition of a macrophage rather than osteoclast fate [26]. Moreover, IL-1ß and other inflammatory signals can increase miR-155 levels via an AP-1 element in its promoter [27].

In melanoma cell lines LB2201-MEL and LB2259-MEL, we observed an upregulation of miR-155 upon treatment with IL-1ß. At 4h this increase reached 2.7-fold and 3.6-fold, and at 24h it reached 8.0-fold and 4.2-fold in LB2201-MEL and LB2259-MEL, respectively (Fig 1B). In contrast, we did not observe any significant upregulation of the other five miRNAs that have been described to target MITF (i.e. miR-96, miR-137, miR-148, miR-340 and miR-182).

### miR-155 targets MITF-M in melanoma cells and reduces the expression of MITF-M target genes

**Fig 2:**
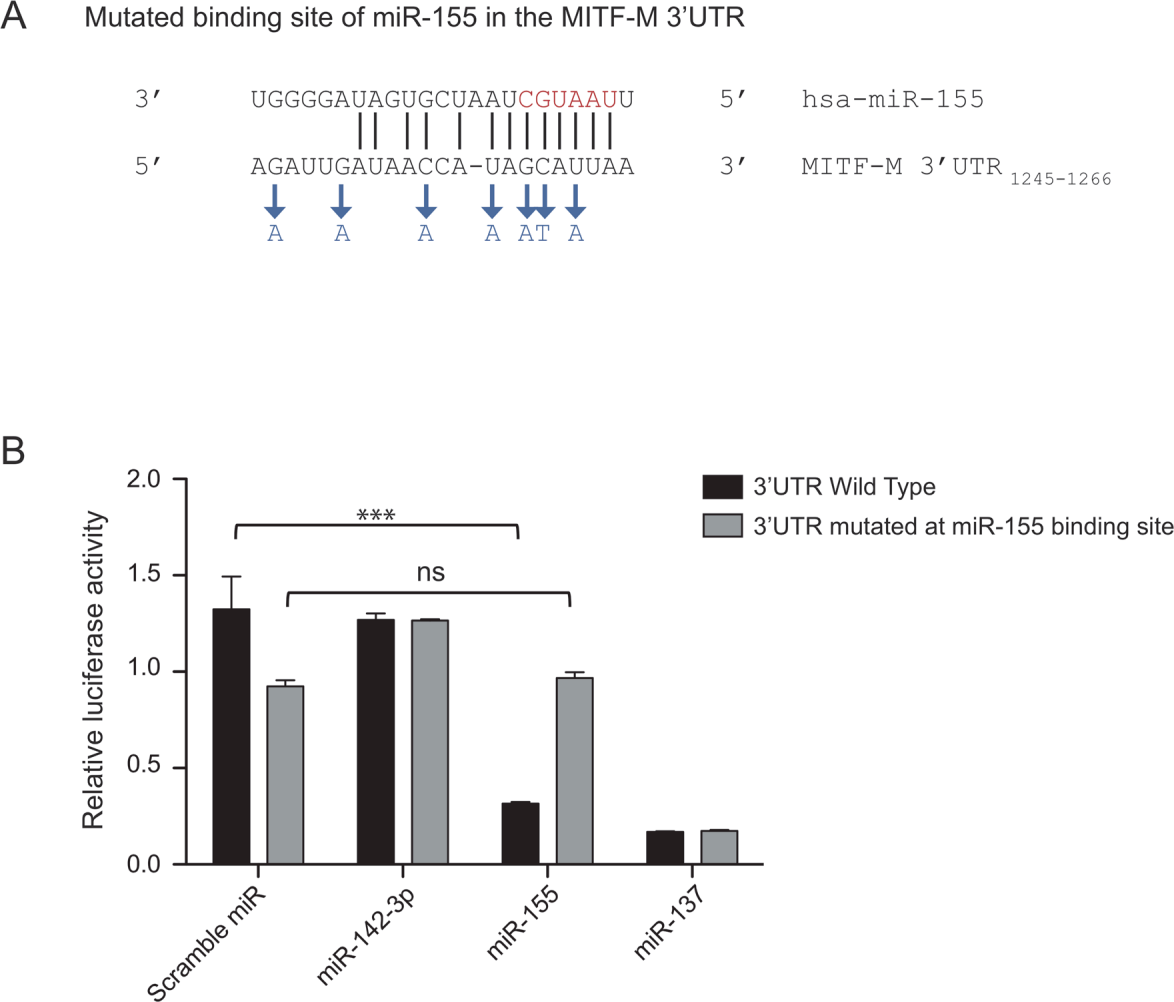
miR-155 targets MITF-M 3’UTR in HEK-293T cells. (**A**) Region of the MITF-M 3’UTR where miR-155 is predicted to bind (subscript numbers indicate the position relative to the first nucleotide after the STOP codon). Seed region of the miRNA sequence is in red. Optimal alignment between MITF-M 3’UTR and miR-155 was predicted by the miRanda software. Blue letters indicate nucleotides in the MITF-M 3’UTR that were substituted by targeted mutagenesis.(**B**) HEK-293T cells were co-transfected with the reporter plasmid psiCHECK-2 containing the 3’UTR of MITF-M wild type (black) or mutated for 7 nucleotides (grey) and the indicated miRNAs mimics (10 nM). The reporter plasmid contains the 3’UTR cloned downstream of the Renilla luciferase gene and the Firefly luciferase gene to take into account the transfection efficiency. Data presented are the normalized ratios of Renilla to Firefly activities (means of triplicates ± SD) and are representative of 3 independent experiments. miR-137 is known to target MITF-M 3’UTR [22] whereas miR-142-3p is an irrelevant miRNA targeting GARP [28].

We next wanted to verify if miR-155 was able to target the MITF-M isoform in melanoma cells. To this end, we cloned the 3kb 3’UTR of MITF-M downstream of an open reading frame coding for Renilla luciferase. Bioinformatics predictions with the miRanda software suggested a single binding site for miR-155 in the 3’UTR of MITF (Fig 2A). When we co-transfected the plasmid containing the wild type 3’UTR with a miR-155 mimic in HEK-293T cells, we observed a 4-fold repression of the luciferase activity compared to a control condition with a scramble mimic or an unrelated miRNA (miR-142-3p) [28]. miR-137 was used as a positive control (Fig 2B). In order to determine if miR-155 directly targets the MITF-M mRNA, we mutated 7 nucleotides in the predicted miRNA binding site within the 3’UTR (Fig 2A). As expected, miR-155 was not able to decrease the Renilla luciferase activity when we transfected the mutated 3’UTR (Fig 2B). Notably, the positive control miR-137 was still able to reduce the Renilla luciferase activity produced from the mutated 3’UTR, suggesting that the introduced mutations do not interfere with the function of other miRNAs that target binding sites elsewhere in the 3’UTR.

Taken together, our results confirm that the original sequence corresponding to the mutated nucleotides is required for miR-155 binding, and that only this single site in the MITF 3’UTR is targeted by miR-155.

**Fig 3:**
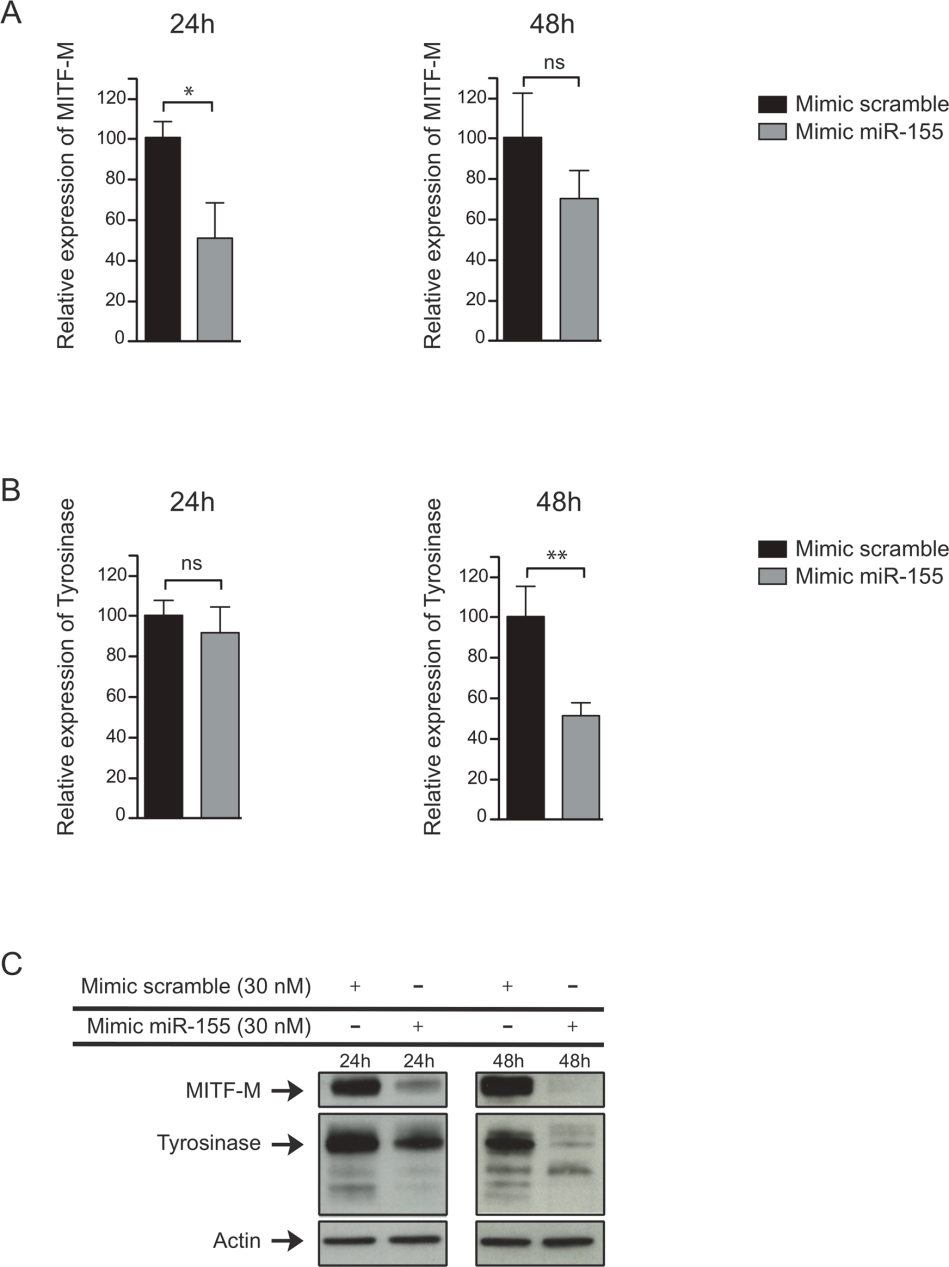
miR-155 downregulates MITF-M in LB2201-MEL melanoma cell line. (**A**) MITF-M and (**B**) Tyrosinase expression was analyzed by quantitative RT-PCR in melanoma cell line LB2201-MEL 24h and 48h after the transfection with a mimic of miR-155 (30 nM). ß-actin expression was used to normalize MITF-M expression (means ± SD for 3 independent experiments). (**C**) MITF-M and Tyrosinase protein expression was analyzed by Western Blot in the same cell line 24h and 48h after the transfection with the mimic of miR-155 (30 nM) (1 of 3 independent experiments).

Then, to test whether miR-155 was able to target endogenous MITF-M transcripts, we transfected a miR-155 mimic in the melanoma cell line LB2201-MEL. We then evaluated the expression of MITF-M and its target gene tyrosinase at the mRNA and protein levels. On RNA level, we observed a decrease in the abundance of MITF-M mRNA after 24h and 48h (Fig 3A) followed by a decrease of tyrosinase mRNA (Fig 3B). This repression of MITF-M and tyrosinase expression was even stronger at the protein level, in particular at 48h when both proteins were almost undetectable (Fig 3C).

Altogether, our results confirm that miR-155 is able to target a single binding site on the 3’UTR of MITF-M, which leads to a strong reduction of the abundance of MITF-M and tyrosinase proteins.

### Endogenous miR-155 contributes to the repression of MITF-M in the presence of IL-1ß

**Fig 4:**
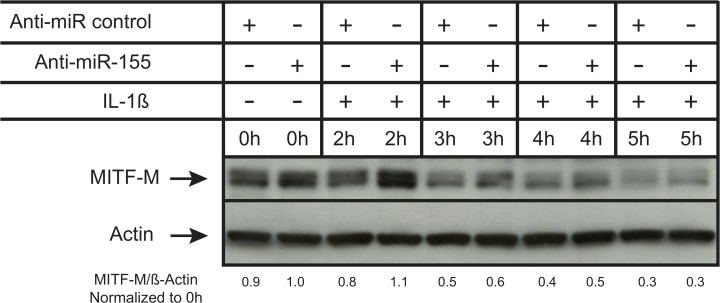
The repression of MITF-M by IL-1ß can be prevented at an early time point by the transfection of an AntagomiR specific to miR-155. LB2201-MEL cells were transfected and incubated for 24h with an AntagomiR specific for miR-155 or a control AntagomiR (150 nM) and then treated with IL-1ß (10 ng/ml) for 2h, 3h, 4h or 5h. This experiment is representative of 3 independent experiments.

To confirm that endogenous miR-155 contributes to the repression of MITF-M expression by IL-1ß, we used antisense inhibitors specific for miR-155. First, we transfected LB2201-MEL cells with either an inhibitor of miR-155 (AntagomiR anti-miR-155) or a control inhibitor. After 24h, we treated the cells with IL-1ß and analyzed the abundance of MITF-M protein 2h, 3h, 4h or 5h later by Western Blot. The repression of MITF-M expression induced by IL-1ß appeared to be delayed in the presence of the anti-miR-155, with the strongest effect after 2h of treatment with IL-1ß (Fig 4). This indicates that endogenous miR-155 contributes to the IL-1ß-induced downregulation of MITF-M. However, the lack of effect of anti-miR-155 at later time points suggests that the inhibitor might not be sufficiently effective at high miR-155 levels observed at later time points. Alternatively, there might be other mechanisms that are more important for IL-1ß-induced downregulation of MITF-M at later time points.

### Downregulation of MITF-M by IL-1ß is the result of the action of miR-155 and of a reduction in MITF-M transcription

To address the question whether other mechanisms contribute to the downregulation of MITF-M by IL-1ß, we chose a qPCR approach. To distinguish the effect of IL-1ß on mRNA transcription from an effect on mRNA stability, we performed quantitative PCR for the unspliced precursor transcript of MITF-M as well as for the mRNA transcript.

**Fig 5:**
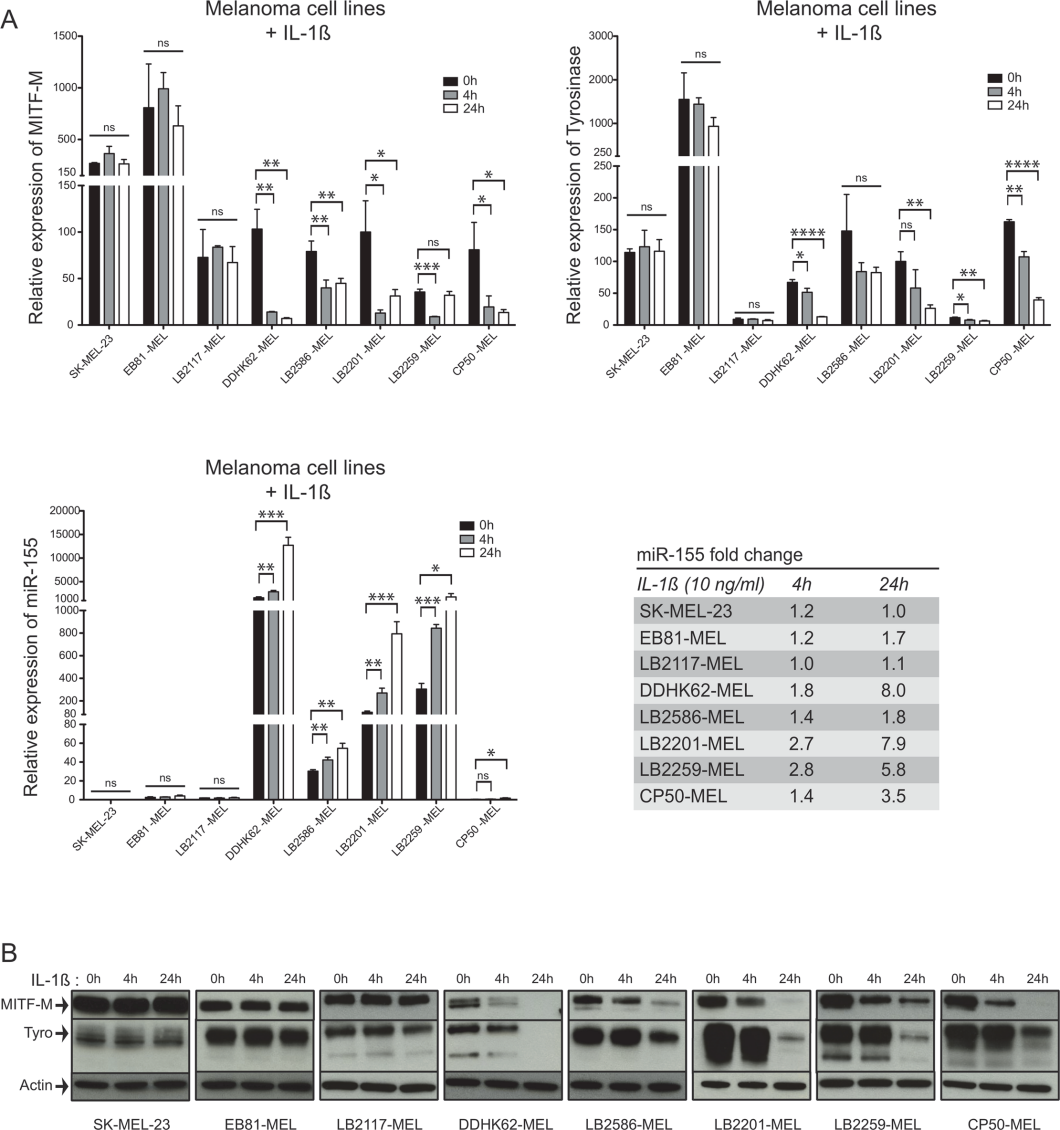
Inverse correlation between the expression of MITF-M and that of miR-155 in melanoma cell lines incubated with IL-1ß. (**A**) The expression of MITF-M, Tyrosinase and miR-155 was analyzed by quantitative RT-PCR in 8 melanoma cell lines incubated with IL-1ß (10 ng/ml) for 4h or 24h. The results were normalized to ß-actin expression for MITF-M and Tyrosinase and to RNU44 for miR-155 (means ± SD for 3 independent experiments). (**B**) The expression of MITF-M and Tyrosinase proteins was analyzed by Western Blot in the same samples (1 of 3 independent experiments).

In the cell lines that show upregulation of miR-155 by IL-1ß our results suggest that both reduced mRNA transcription and mRNA destabilization occur (S1 Fig). We also analyzed one cell line that shows no increase in miR-155 upon treatment, CP50-MEL (Fig 5). As expected, in this cell line only an effect on transcription was observed. Unfortunately, we were not able to delineate the cis-element that mediates IL-1ß induced-repression of MITF-M transcription in reporter assays (data not shown). This suggests that this effect is mediated by an element outside of the core promoter.

To get an impression of the relative contribution of miR-155 and transcriptional repression, we next wanted to broaden our experiments by analyzing more melanoma cell lines after treatment with IL-1ß for 4h or 24h. As expected, the cell line SK-MEL-23 that lacks the receptor for IL-1ß [19], did not show any changes in miR-155, MITF-M and tyrosinase expression (Fig 5). Similarly, cell lines EB81-MEL and LB2117-MEL did not exhibit any repression of MITF-M or tyrosinase nor any upregulation of miR-155 expression. However, in the cell lines DDHK62-MEL, LB2586-MEL, LB2201-MEL and LB2259-MEL, downregulation of MITF-M and tyrosinase (at the mRNA and protein levels) and upregulation of miR-155 were clearly observed after treatment with IL-1ß for 4h and 24h (Fig 5). The increase in miR-155 reached between 1.4- and 2.8-fold after 4h, and between 1.8 and 8.0-fold after 24h of treatment (Fig 5).

### Expression levels of MITF-M and miR-155 are inversely correlated in a mouse model of inducible melanoma

Immune evasion of melanoma cells represents an obstacle to the function of CTL and to the clinical efficiency of cancer immunotherapy. To test whether miR-155 might contribute to the downregulation of MITF-M and melanocyte differentiation antigens in melanoma *in vivo*, we analyzed samples from a mouse model of malignant melanoma. We chose to utilize the Tirp transgenic mouse model, which has been previously developed in our laboratory [21]. In this inducible model of melanoma, loss of the tumor suppressor INK4A/ARF and the concomitant expression of the H-RAS (G12V) oncogene and of the natural “cancer-germline gene” P1A, occur specifically in melanocytes [21].

**Fig 6:**
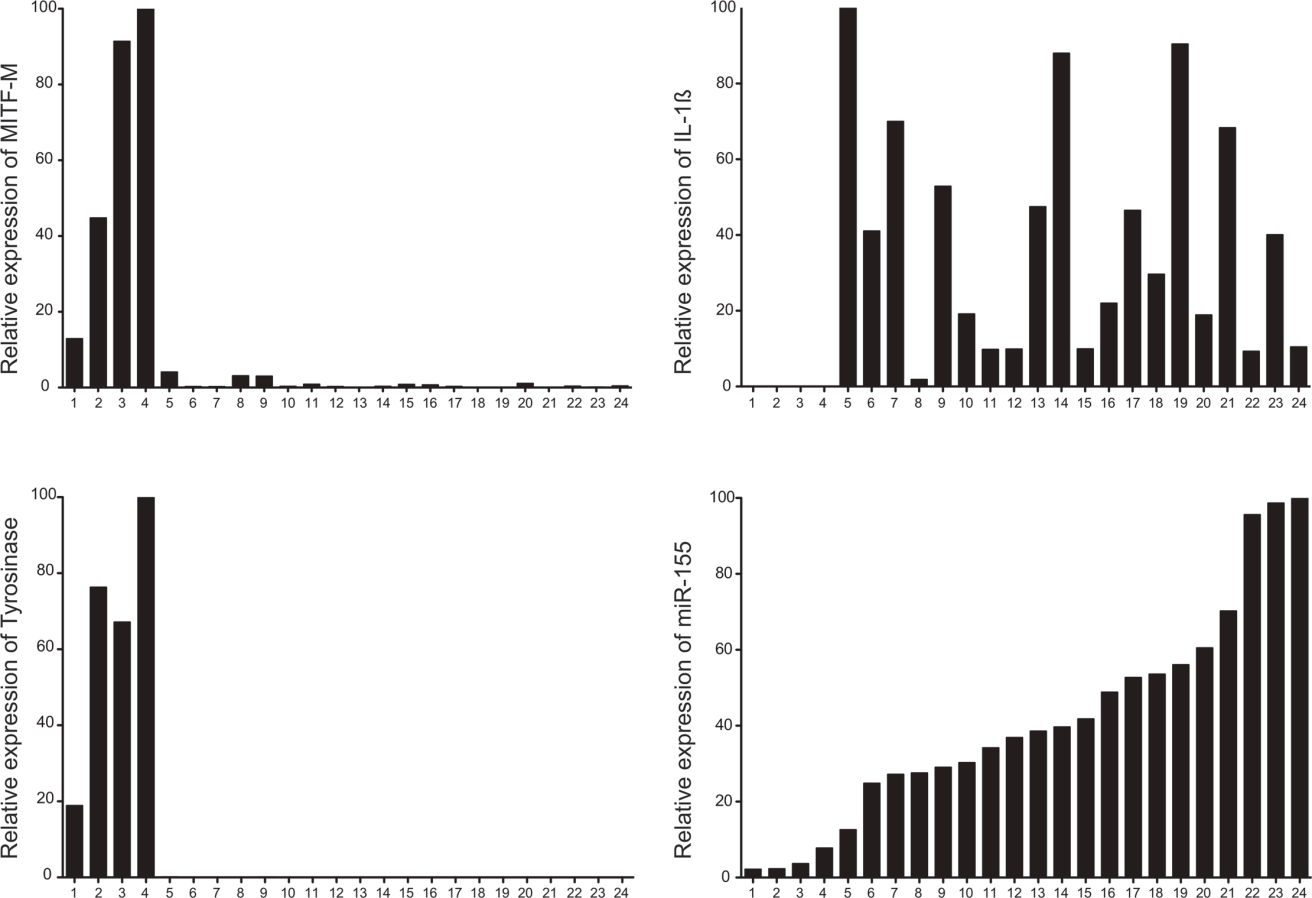
In mouse melanoma samples, there is an anti-correlation between the expression of MITF-M or Tyrosinase and that of IL-1ß or miR-155. 24 tumor samples were collected from 23 Tirp10B mice [21] and the expression of MITF-M, Tyrosinase, IL-1ß and miR-155 was analyzed by quantitative RT-PCR. ß-actin was used as an internal control for MITF-M, Tyrosinase and IL-1ß whereas U6 was used as an internal control for miR-155. Taken together, our results suggest that the inverse correlation between the expression levels of miR-155 on the one side, and MITF-M and tyrosinase on the other side is also observed *in vivo*.

We collected 24 tumor samples from 23 mice and analyzed the expression of MITF-M, tyrosinase, IL-1ß and miR-155 by quantitative RT-PCR using RNA extracted from total tumor tissue. We found that 4 out of 23 samples expressed MITF-M and tyrosinase, did not express IL-1ß and expressed weakly miR-155 (Fig 6). On the other hand, 19 out of 23 melanoma samples did not express MITF-M and tyrosinase or expressed low levels of MITF-M but had high levels of expression of miR-155. All these 19 tumor samples showed expression of IL-1ß to varying degree, suggesting that this cytokine could contribute to the upregulation of miR-155. We should note, however, that other inflammatory cytokines share the downstream signaling cascade with IL-1ß and might very well contribute to the expression of miR-155 in these samples (Fig 6).

## Discussion

The objective of this study is to understand the molecular mechanisms by which IL-1ß regulates MITF-M in melanoma. We show here that miR-155 is upregulated following the treatment with IL-1ß in several melanoma cell lines and can reduce the expression of MITF-M both at the mRNA and—even more efficiently—at the protein level. This effect can be partially prevented by anti-sense miR-155 inhibitor, suggesting that endogenous miR-155 takes part in the regulation of MITF-M.

The majority of the cell lines were sensitive to IL-1ß. However, not all cell lines where IL-1ß leads to reduced MITF-M expression, show an increase in miR-155. Furthermore, qPCR experiments assessing unspliced precursor RNAs and mRNAs suggest that MITF-M levels are regulated by both destabilization of the mRNA, as well at the level of transcription (S1 Fig).

To understand the mechanism of the transcriptional regulation of MITF-M by IL-1ß, we performed reporter gene assays in the presence and absence of IL-1ß. Using reporter constructs containing 4.5kb and 15kb promoter fragments encompassing the MITF-M transcriptional start site and binding sites for known regulators of MITF-M (e.g. PAX3 and SOX10, [29] [30] [31]), we did not observe any effect of IL-1ß on reporter gene activity in the melanoma cell line LB2259-MEL (data not shown). In further experiments, we tested the enhancer activity of several genomic regions that show binding to several transcription factors in the chromatin immunoprecipitation datasets of the ENCODE consortium (http://www.genome.ucsc.edu/ENCODE/). The first region corresponding to the end of intron 3, exon 4 and the beginning of intron 4 included some sequences that could be targeted by the transcription factors c-Jun and JunD (chr3 69987713–69989931 ref: AC_000135.1). The second region corresponding to the complete intron 4 (chr3 69989863–69997581 ref: AC_000135.1) presented 3 binding sites for the transcription factor CTCF and the third region located 30 kb after the end of the MITF-M 3’UTR could be targeted by 27 transcription factors, including some transcriptional repressors (E2F6, ZBTB7A, HEY1,…) (chr3 70046346–70051387 ref:AC_000135.1). Putative enhancer sequences were cloned downstream of a luciferase reporter and a 2.2kb MITF-M promoter. Again, we did not observe any effect of IL-1ß on the activity of these reporter constructs (data not shown).

While these experiments do not provide evidence that transcriptional regulation of MITF-M by IL-1ß occurs via the tested promoter fragment, they do not formally exclude a direct effect either. PAX3 and SOX10 are two key regulators of MITF-M expression at the transcriptional level. To address whether changes in PAX3 or SOX10 levels might be responsible for the effect of IL-1ß, we performed quantitative RT-PCR and Western Blot analyzes in several melanoma cell lines. As shown in S2 Fig, we did not detect any significant change in the expression of these two transcription factors by IL-1ß.

It therefore seems likely that the repression of MITF-M transcription occurs via another unidentified regulatory region, via regulation of chromatin composition that is inadequately represented in reporter assays or with a more complex mechanism involving the cooperative binding of several transcription factors.

To understand the contribution of miR-155 to MITF-M expression and potentially to the expression of CTL target antigens, we employed a mouse model of melanoma referred as Tirp [21]. We observed a strong negative correlation between MITF-M and tyrosinase on the one side, and miR-155 and IL-1ß on the other side. The tumors induced in those mice are known to present 2 different phenotypes: some are nonpigmented, able to grow fast and kill the mice within one month (they were named “Amela”); others are pigmented, less aggressive and lethal for the mice within three months (designed as “Mela”) [32]. Analysis of these tumors revealed that Amela tumor progression is linked to inflammation, splenomegaly and myeloproliferative disorders. Indeed, inflammatory cytokines like IL-6 and other chemokines like IL-8 are upregulated in the supernatant of Amela compared to Mela tumor cells [32]. Moreover, it has also been shown that the expression of IL-1ß (and small quantities of TNF-α and IL-1α) was induced in the sera of mice bearing Amela tumors. Our results are in agreement with this study. Indeed, the presence of melanin, responsible for the Mela pigmented phenotype, is mainly determined by MITF-M, which upregulates the genes responsible for the melanogenesis, including tyrosinase. This phenotype is not associated with an inflammatory environment, contrary to the Amela nonpigmented tumors. Therefore, it could be interesting to inhibit the expression of miR-155 in Amela tumors (by the use of an Antagomir for example) to evaluate if we could reduce their aggressiveness by redirecting them to a Mela phenotype. Indeed, the reexpression of MITF-M in these tumors could have an influence not only on the production of melanin but also on the proliferation rate and the invasiveness of the tumor cells. Another option to investigate the influence of this miRNA in melanoma progression could be to use the inducible Cre system present in the Tirp model to delete the gene producing miR-155.

Here, we discovered a new mechanism of MITF-M repression in the context of an immune response against melanoma cells. However, we could imagine that the repression of MITF-M by miR-155 also occurs in melanocytes in a context of differentiation, for example. It was already described that miR-155 is involved in a differentiation process by targeting an isoform of MITF [26]. Indeed, inflammatory signals detected by toll-like receptors in murine monocyte progenitors induce their differentiation into active macrophages while their stimulation with the receptor activator of nuclear factor κB ligand (RANKL) and macrophage colony-stimulating factor (M-CSF) favors an osteoclast fate [26]. The signaling pathway induced by RANKL, an osteoblast-derived factor, leads to the upregulation of MITF and PU.1, which are essential transcription factors for the differentiation into osteoclasts while the upregulation of miR-155, induced by inflammatory signals, favors the differentiation into macrophages by targeting MITF and repressing its expression [26]. Moreover, it is known that the expression of MITF-M is precisely regulated during melanocyte differentiation [33]. Melanocytes are derived from a population of pluripotent neural crest cells and the expression of MITF-M in some of those cells is necessary to allow them to differentiate first into melanoblasts and then into melanocytes [33]. Melanoblasts that migrate to the hair follicle can produce 2 different kinds of melanocytes: differentiated, melanin-producing melanocytes that express MITF-M and a second population that has lost the expression of MITF-M and was called”stem cell-like” melanocytes [34]. This second population is mostly quiescent but can be activated and used for melanocyte self-renewal at each hair regeneration cycle [35]. It was shown that the presence of Transforming Growth Factor-ß (TGF-ß) in the melanoblast microenvironment represses the expression of MITF and favors melanoblast differentiation into a “stem cell-like” phenotype [35]. This repression of MITF-M by TGF-ß was also shown in melanoma cells and occurred through several mechanisms including a repression of the protein kinase A (PKA) [36]. However, TGF-ß is also able to directly upregulate miR-155 expression, which can therefore represent another mechanism of MITF-M repression induced by this cytokine [37]. Moreover, we can imagine that other cytokines that regulate MITF-M expression could also be present in the microenvironment and then influence the melanocyte differentiation process. It was shown that IL-1α, IL-6 or TNF-α produced by human keratinocytes or melanocytes themselves could reduce the proliferation and the tyrosinase activity of melanocytes [38]. We also observed a reduction of MITF-M and tyrosinase expression in human normal melanocytes treated with IL-1ß (data not shown). Therefore, for a better understanding of melanocyte differentiation, the expression of miR-155 and inflammatory cytokines including IL-1ß should be investigated during the differentiation of the melanoblasts to check whether they could influence the appearance of one particular phenotype.

Finally, the traditional genetic model of melanoma metastasis proposes that some cells within the primary tumor acquire irreversible pro-metastasis mutations and then migrate to form metastases genetically different from the initiating tumor cells [34]. However, an alternative model suggests that the activation of some pathways by the microenvironment rather than genetic mutations could explain the changes in the cell phenotype that promote metastasis from the primary tumor site [34] [39]. Indeed, melanoma are known to be heterogeneous tumors and changes in the microenvironment like hypoxia or inflammation could favor the switch, in some tumor cells, from a proliferative phenotype where the cells express MITF-M to an invasive phenotype where they do not express MITF-M and acquire the potential to invade and form metastasis. Moreover, once those cells have migrated to another site, they could switch back to a proliferative phenotype if the microenvironment is suitable [34] [39] [40] [41]. This “phenotype switching” explains why the melanoma cells are so difficult to eradicate. Because treatments like chemotherapy target proliferative cells but spare cells exhibiting an invasive and slow growth phenotype, those cells could next reconstitute a heterogeneous population by switching back to a proliferative state.

Immunotherapy also targets MITF-positive differentiated cells expressing specific antigens. Therefore, to improve anti-melanoma therapy, resistant subpopulations should be redirected to a sensitive phenotype with a treatment that is efficient independently of the presence of mutations frequently found in melanoma cells [42]. The potential of this approach has been shown in the use of 3-O-(3,4,5-trimethoxybenzoyl)-(-)epicatechin (TMECG) in metastatic melanoma therapy. This prodrug is activated by tyrosinase, which leads to the inhibition of the dihydrofolate reductase (DHFR) essential enzyme. The activation of this prodrug being tyrosinase dependent, only the cells expressing this enzyme will be killed. However, the combination of TMECG with methotrexate (MTX), which is able to upregulate MITF-M and therefore tyrosinase expression in melanoma cells, leads to the death of the tumor cells regardless of the mutation status of BRAF, MEK or p53 [42]. Moreover, this kind of therapy is very advantageous because all the melanoma cells treated with MTX are forced to switch into a MITF-M and tyrosinase-positive phenotype and become sensitive to TMECG.

In conclusion, here for the first time we showed that miR-155 targets MITF-M in an inflammatory microenvironment. Inhibition of miR-155 or inhibition of the inflammatory pathways might represent an additional therapeutic approach to the conventional chemotherapy and immunotherapy for melanoma.

## Supporting Information

S1 Fig Repression of the transcription versus destabilization of MITF-M in melanoma cell lines incubated with IL-1ß. Melanoma cell lines were incubated with IL-1ß for 4h or 24h and the expression of MITF-M was analyzed by quantitative RT-PCR with 2 different pairs of primers. The “inter-exon” primers located in the first and second exon of MITF-M allowed us to evaluate the total content of mature MITF-M mRNA that could be reduced either by the repression of the transcription or by the destabilization of the transcript. The “intron 1” primers were located in the first intron of MITF-M and detected the unspliced mRNA before destabilization by miRNAs, allowing us to evaluate only the repression of the transcription. We performed quantitative RT-PCR with those 2 pairs of primers in 5 cell lines sensitive to IL-1ß and observed that all of them except CP50-MEL showed a stronger repression of MITF-M expression with the “inter-exon” primers than with the “intron 1” primers. Because a repression of MITF-M expression was observed already with the “intron 1” primers, this suggest that the mRNA abundance is reduced both by a repression of the transcription and by a destabilization of the transcripts. Moreover, destabilization of the transcripts by miR-155 could be detected by the “inter-exon” primers but because of the miRNA mechanism of action, it should be more effective at the protein level. Since CP50-MEL shows the same level of repression of MITF-M expression with the two pairs of primers, the repression of MITF-M on this cell line seems to occur only by a repression of the transcription of MITF-M. In both quantitative RT-PCR, the expression of MITF-M was normalized to ß-actin (means ± SD for 3 independent experiments) and then to the expression of MITF-M in each control condition (100% at t = 0).
(TIF)

S2 Fig The expression of PAX3 and SOX10 is not affected by IL-1ß. The expression of PAX3 (**A**) and SOX10 (**B**) was analyzed by quantitative RT-PCR and Western Blot in 8 melanoma cell lines incubated with IL-1ß (10 ng/ml) for 4h or 24h. The results were normalized to ß-actin expression (means ± SD for 3 independent experiments).
(TIF)
