# Supplementary figures and images for: microRNA-155, Induced by Interleukin-1ß, Represses the Expression of Microphthalmia-Associated Transcription Factor (MITF-M) in Melanoma Cells
Source: PLoS One. 2015 Apr 8;10(4):e0122517. doi: 10.1371/journal.pone.0122517 (PMC4390329; doi:10.1371/journal.pone.0122517)

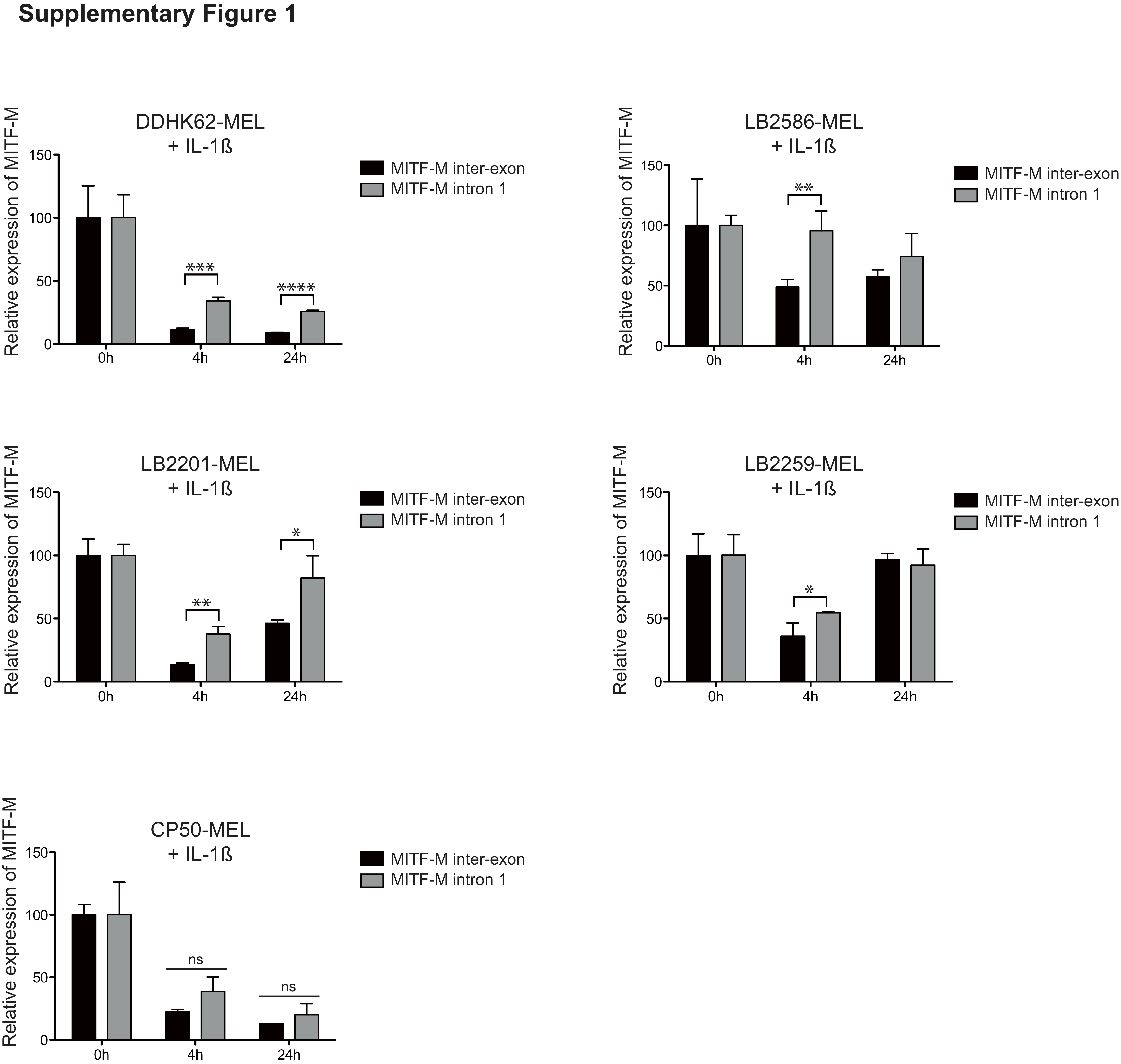

Supplement: S1 Fig — Melanoma cell lines were incubated with IL-1ß for 4h or 24h and the expression of MITF-M was analyzed by quantitative RT-PCR with 2 different pairs of primers. The “inter-exon” primers located in the first and second exon of MITF-M allowed us to evaluate the total content of mature MITF-M mRNA that could be reduced either by the repression of the transcription or by the destabilization of the transcript. The “intron 1” primers were located in the first intron of MITF-M and detected the unspliced mRNA before destabilization by miRNAs, allowing us to evaluate only the repression of the transcription. We performed quantitative RT-PCR with those 2 pairs of primers in 5 cell lines sensitive to IL-1ß and observed that all of them except CP50-MEL showed a stronger repression of MITF-M expression with the “inter-exon” primers than with the “intron 1” primers. Because a repression of MITF-M expression was observed already with the “intron 1” primers, this suggest that the mRNA abundance is reduced both by a repression of the transcription and by a destabilization of the transcripts. Moreover, destabilization of the transcripts by miR-155 could be detected by the “inter-exon” primers but because of the miRNA mechanism of action, it should be more effective at the protein level. Since CP50-MEL shows the same level of repression of MITF-M expression with the two pairs of primers, the repression of MITF-M on this cell line seems to occur only by a repression of the transcription of MITF-M. In both quantitative RT-PCR, the expression of MITF-M was normalized to ß-actin (means ± SD for 3 independent experiments) and then to the expression of MITF-M in each control condition (100% at t = 0). (TIF) [file pone.0122517.s001.tif]

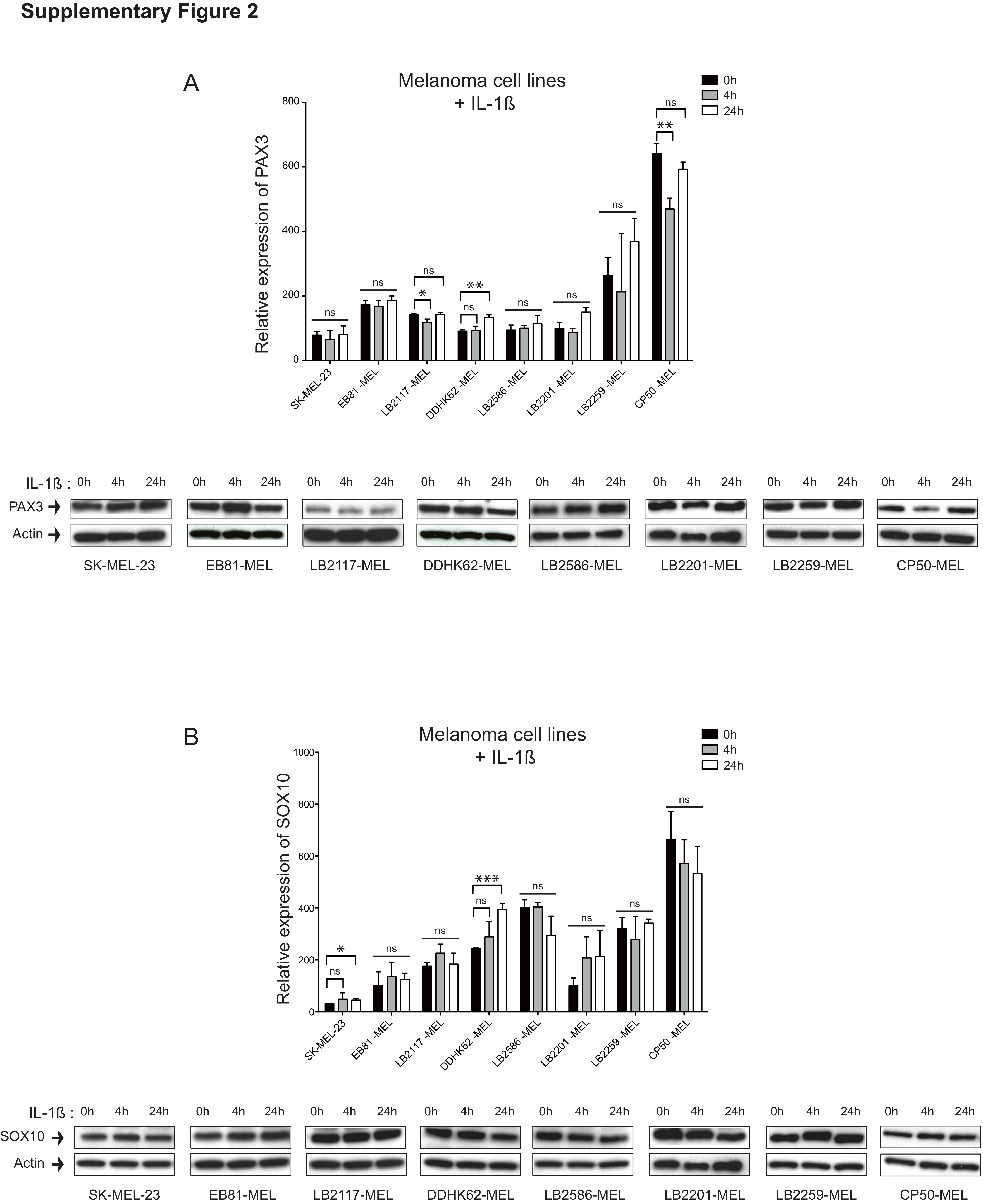

Supplement: S2 Fig — The expression of PAX3 (A) and SOX10 (B) was analyzed by quantitative RT-PCR and Western Blot in 8 melanoma cell lines incubated with IL-1ß (10 ng/ml) for 4h or 24h. The results were normalized to ß-actin expression (means ± SD for 3 independent experiments). (TIF) [file pone.0122517.s002.tif]
